# The Relationship Between Procrastination, Learning Strategies and Statistics Anxiety Among Iranian College Students: A Canonical Correlation Analysis

**Published:** 2012

**Authors:** Shahrum Vahedi, Farahman Farrokhi, Farahnaz Gahramani, Ali Issazadegan

**Affiliations:** 1 Associate professor, English Dept., University of Tabriz.; 2 Master of art in curriculum, Assistant professor, psychology Department, University of Urmia.

**Keywords:** learning strategies, Procrastination, Self-regulation, statistics anxiety

## Abstract

**Objective:** Approximately 66-80%of graduate students experience statistics anxiety and some researchers propose that many students identify statistics courses as the most anxiety-inducing courses in their academic curriculums. As such, it is likely that statistics anxiety is, in part, responsible for many students delaying enrollment in these courses for as long as possible. This paper proposes a canonical model by treating academic procrastination (AP), learning strategies (LS) as predictor variables and statistics anxiety (SA) as explained variables.

**Methods:** A questionnaire survey was used for data collection and 246-college female student participated in this study. To examine the mutually independent relations between procrastination, learning strategies and statistics anxiety variables, a canonical correlation analysis was computed.

**Results**: Findings show that two canonical functions were statistically significant. The set of variables (metacognitive self-regulation, source management, preparing homework, preparing for test and preparing term papers) helped predict changes of statistics anxiety with respect to fearful behavior, Attitude towards math and class, Performance, but not Anxiety.

**Conclusion**: These findings could be used in educational and psychological interventions in the context of statistics anxiety reduction.

## Introduction

Anxiety affects many people in many situations. In academic settings, anxiety can have important negative effects on cognitive functioning, learning, performance and success (cited in 1). Anxiety has been found to be one of the most prevalent attitudinal problems associated with statistics courses (1). Researchers have clustered variables that affect statistics anxiety around three main factors: personality-related, course-related, and person-related (environmental). Herein, procrastination and organization play a large role in statistics anxiety. 

Many students tend to experience high levels of statistics anxiety when confronted with statistical ideas, problems, or issues, instructional situations, or evaluative situations (2). Anxiety toward statistics affects learning and is the best predictor of achievement in research methodology and statistics courses (3).

 “Causes'' of statistics anxiety are usually clustered around three major factors: dispositional, situational, and environmental. Dispositional factors include psychological and emotional characteristics such as attitudes toward statistics, perceptions, self- concept, and learning styles. Situational factors of statistics anxiety are immediate factors that result from statistics courses and include, but not limited to, statistics teachers, nature of statistics courses, lack of feedback from statistics instructors, pace of statistics instruction, and statistical terminology. Environmental causes are factors that “have affected the individual prior to the statistics course” and include gender, age, ethnicity, academic major, and previous mathematics experiences Gender is one of the most widely investigated environmental variables in statistics anxiety research. In general, women have been reported to encounter more difficulties in quantitative areas. Similarly, Stroup and Jordan, Zeidner, and Onwuegbuzie reported that women experienced higher levels of statistics anxiety than did men in their studies (3).

Furthermore, statistical anxiety has been shown to be related to increased levels of academic procrastination (2). However, procrastination may be a viable, though likely ineffective cognitive strategy for some students. Onwuegbuzie (2) observed that in a sample of graduate students enrolled in an introductory level educational research course, academic procrastination was related to a fear of failure and task aversiveness. Additionally, this relationship predicted several aspects of statistics anxiety including worth of statistics, interpretation anxiety, test and class anxiety, computational self-concept, fear of asking for help, and fear of the statistics instructor. Procrastination has also been shown to be related to interpretation anxiety, fear of asking for help, and fear of statistics teachers when considered in combination with trait anxiety and intrapersonal perfectionism (4). However, its relation to other learning strategies in the prediction of statistics anxiety is unknown. Academic procrastination refers to the postponement of academic goals to the point where optimal performance becomes highly unlikely. Solomon and Rothblum (cited in 5) found that 46% of their undergraduate participants procrastinated on writing term papers, with 27.6% doing so when studying for exams, and 30.1% when reading weekly assignments. Onwuegbuzie reported similarly high levels of academic procrastination with graduates, across the same three tasks of 41.7%, 39.3%, and 60.0%, respectively (2).

 Procrastination has also been shown to be related to interpretation anxiety, fear of asking for help, and fear of statistics teachers when considered in combination with trait anxiety and intrapersonal perfectionism (4). However, its relation to other learning strategies in the prediction of statistics anxiety is unknown.

The extent to which students cognitively process academic material effectively depends on the learning strategies that are employed. However, studies investigating statistics anxiety and learning strategies are limited. Investigating the ways in which these two constructs are related makes sense considering the cognitive aspect of anxiety thought to persist in academic-type anxiety. For example, learning strategies focused on the learner's self-directed process of maintaining thoughts and behaviors to reach one’s academic goals have been associated with an increase in computational skills, mathematical achievement, and comprehension (6). 

Previous studies have shown that procrastination and learning strategies (rehearsal and organization) all jointly and positively predict interpretation anxiety and test and class anxiety. If a student has not been keeping up with his assignments or reading, he may likely use rehearsal and organization strategies as an ineffective means of trying to catch up, resulting in higher levels of anxiety when understanding concepts in class and on tests. With these findings, one might suggest that for men with statistics anxiety, assisting them with strategies addressing procrastination might be helpful. Such things as daily class assignments or in-class assignments may help them stay current in their study habits, making them less reliant on ineffective learning strategies and more open to asking for help when it is needed (6).

Overall, procrastination was positively related to all aspects of statistics anxiety where the other learning strategies were negatively related to statistics anxiety. However, the most useful relationships are between procrastination, time and study environment, metacognitive self-regulation, and help seeking as they predicted worthlessness of statistics, lack of computational self-concept, and fear of asking for help. Again, procrastination is negatively related to these variables. Regarding the predictor variables, interpretation anxiety and test and class anxiety were positively related to each other and the primary contributors to the predictor synthetic variable. This was supported by both the function and structure coefficients. Overall it can be said that the more procrastination, rehearsal, and to some extent elaboration and organization strategies women use, the more likely they are to experience interpretation anxiety, and test and class anxiety.

Women trend to experience more test anxiety, statistics anxiety, and math anxiety then men (6).

To date no study appears to have investigated the link between academic procrastination, learning strategies and statistics anxiety among female students, which was the purpose of the present study. Whereas academic procrastination has been related positively to generalize and specific kinds of anxiety such as test anxiety and social anxiety (5), it was hypothesized that academic procrastination would be positively related to statistics anxiety. In addition, we hypothesized that learning strategies is a negative predictor of statistics anxiety.

## Materials and Methods

Participants were 246 undergraduate female students from different disciplines of human sciences, enrolled in entry-level statistics courses of Tabriz University in Iran, who voluntarily participated in the study and signed the consent form. College research examination Board approved the research protocol. All participants were recruited opportunistically using a cluster-sampling technique initiated by three data collectors. All participants completed paper-and-pencil versions of the questionnaire anonymously, and returned the questionnaires to their contact person. All data were treated confidentially, and participants were provided with a debrief sheet following completion. All participants took part on a voluntary basis and were not remunerated for participation.

*Statistics Anxiety Measure Earp* (7). The 43 items of this scale are rated on a 5-point scale ranging from 1= strongly disagree to 7= strongly agree (higher scores reflect greater statistics anxiety; see Appendix A for the list of items). SAM comprises five discrete subscales: anxiety, performance, and attitude towards class, attitude towards math, and fearful behavior. The English versions of the scale show a multidimensional structure for student, and have good construct, discriminate validities (7).The internal consistent reliability of overall scale (α = 0.93) as well as sub-scales generally ranged from high to excellent (α = 0.82– 0.95). The Persian version of the SAM was developed using the standard back-translation technique (8).The first author initially translated the SAM into Persian and an independent translator unaffiliated with the study then translated this version back into English. Minor differences that emerged during this process were resolved between translators.

*Procrastination Assessment Scale– Students* (*PASS)*; Solomon & Rothblum, (cited in 5). students rated the extent to which they procrastinate in three academic areas (preparing homework, preparing for test and preparing term papers) and the extent to which procrastination in these areas is a problem for them, using five-point scales with endpoints labeled 1 (never procrastinate) and 5 (always procrastinate) for the prevalence items and 1 (not at all a problem) and 5 (always a problem) for the perceived problem items. Responses are summed across the 27 items, with higher scores indicating greater procrastination. Jowkar and Agapoor (9) reported an alpha coefficient 0.91.

*Motivated strategies for learning questionnaire* *(MSLQ).* The MSLQ is an 81-item questionnaire designed to assess one’s motivational orientations and learning strategies. For the purposes of the current study, the learning strategies subscales were used. These consist of 31 self-report items measured on a 7-point Likert scale. The subscales and the internal consistency coefficient alpha estimates for the current sample are as follows: rehearsal (reciting or naming items from a list to be learned; 0.66), elaboration (such as paraphrasing, summarizing, creating analogies, and generative note-taking; 0.78), organization (selecting appropriate information and constructing connections among the information to be learned; 0.68), critical thinking (ability to report applying previous knowledge to new situations in order to solve problems; 0.83), metacognitive self-regulation (awareness, knowledge, and control of cognition; 0.78), time and study environment (manage time and study environments, planning, and scheduling; 0.71), effort regulation (ability to control effort and attention in the face of distractions and uninteresting tasks; 0.68), peer learning (collaborating with peers; 0.67), and help seeking (knowing when it is time to get help from peers, instructor or tutor; 0.67) (10).

*Data analysis*

The major analytical procedure used in this study was canonical correlation. This multivariate analysis is used to examine the relationship between two sets of measures, when each set contains two or more variables or subscales (11). Canonical correlation was utilized to identify a combination of Statistics Anxiety and learning strategies dimensions that might predict a combination of procrastination dimensions (i.e. preparing homework, preparing for test and preparing term papers). Specifically, canonical correlation analyses generate a number of varieties equal to the number of variables in the smallest set, with each successive variate being orthogonal to the previous one and explaining successively less of the variation between the two sets of variables. In other words, variables “are combined to produce, for each side, a predicted value that has the highest correlation with the predicted value on the other side” (12).

The square of the canonical correlation “expresses the proportion of variance in each composite that is related to the other composite of the pair” (14). The two weighted composites are jointly referred to as a canonical variant. The variance explained by a canonical variant may be partially led from the original correlation matrix and a second canonical variant may be formed from the residuals. This canonical variant will be orthogonal to the first canonical variant and will always explain less of the variance than the first variate. The process may be repeated until a non-significant canonical variant is found, or when the number of variants is equal to the number of variables in the smaller set. Canonical correlation analysis was employed to answer the following questions: 1) how many reliable canonical variant pairs are there in the combined SA, AP and LS data set? b) Along how many dimensions are the 5 SA subscales related to the 3 AP and 5 LS scales? c) How are the dimensions that relate the SA subscales and the AP and LS scales to be interpreted? d) How strong is the correlation between variants in a pair?

## Results

The objective of canonical correlation analysis is to determine a linear combination relationship between the explained and predictor variables such that their correlation is subjected to maximization. Table1 presents part of the correlation matrix from which the canonical roots were generated. It can be seen that the statistics anxiety factor was positively related to Procrastination, but the learning strategies factor was negatively associated with it.

**Table 1:** Pearson product- moment correlations of procrastination measures and the statistics anxiety and learning strategies dimensions

Statistics Anxiety and learning strategies factors	Statistics Anxiety				
	Attitude towards math	Attitude towards class	Anxiety	Fearful behavior	Performance
Procrastination measure:					
1) Preparing homework	-.21^**^	-.12	-.14^*^	-.16^*^	-.26^**^
2) Preparing term papers	-.18^**^	-.07	-.15^*^	-.09	-.20^**^
3) Preparing for test	-.10	-.08	-.09	-.15^*^	-.14^*^
learning strategies:					
1) Cognitive metacognitive	-.24^**^	-.29^**^	-.10	-.23^**^	-.23^**^
2) Source management	-.09^**^	-.01	-.01	-.10	-.14^**^
3) regulation	-.01	-.05	-.11	-.06	-.03
4) Cooperative in learning	-.01	-.03	-.04	-.06	-.02
5) Research help	-.04	-.03	-.04	-.05	-.02

* p < .05

** p < .001

Table 2 shows the canonical functions of this study. These canonical functions capture all the correlations between the explained and predictor variables. Two canonical functions were reported to be significant at P<.01 that is functions one and two.

**Table 2:** Results of canonical correlations

Root number	Eigenvalue	Wilks lambda	F	df.	Canonical correlation	Significance of F-test
1	.20	.69	2.23	40	.41	.001
2	.13	.83	1.59	28	.34	.03
3	.04	.94	.77	18	.19	.74
4	.02	.97	.51	10	.14	.88
5	.001	.99	.06	4	.03	.99

Table 3 shows the canonical loadings of these two functions. The redundancy indexes report as 15.20 for the first function and 17.84 for the second function. The solution of the model is further elaborated below.

Table 3 presents data pertaining to the first and second canonical root. This table provides both standardized function coefficients and structure coefficients. Using a cutoff correlation of 0.3 (14), the structure coefficients pertaining to the first canonical function revealed that five variables from the SA set were meaningfully correlated with the first canonical variant (see Table 2), namely attitude towards math (r = 0.81), attitude towards class (r = 0.58), anxiety (r = 0.30), fearful behavior (r = 0.86), and performance (r = 0.56). Three of the eight variables in the AP and LS set were correlated with the first canonical variant (function), namely preparing Homework (r = 0.46), Preparing for paper (r = 0.36), and Metacognitive self-regulation (r = - 0.76). In addition, metacognitive self-regulation is significantly and negatively correlated to the above significant linear combination of explained variables. With respect to AP and LS, metacognitive self-regulation made a substantial contribution, and preparing for paper a low contribution. 

Two variables from the SA set were correlated with the second canonical variant, namely attitude towards class (r = -0.51), and performance (r = 0.64). The following variables from the AP and LS set were correlated with the second canonical variant: preparing Homework (r = 0.59), Preparing for test (r = 0.63), Metacognitive self-regulation (r = -0.34), and Source management (r = -0.72).

The square of the structure coefficient indicated that each of variables Function above explained 19%, and 08% of the variance, respectively.

**Table 3:** Canonical solution for first and second function: relationship between statistics anxiety subscales, learning strategies dimensions and procrastination subscales

Variable	Canonical loadings				
	Function 1		Function 2		
Explained variables (SA)	Standardized coefficient	Structure coefficient	Standardized coefficient	Structure coefficient	
Attitude towards math	-.53*	-.81	-.16	.09
Attitude towards class	-.25	-.58	.98*	.51*
Anxiety	-.15	-.30	.13	.23
Fearful behavior	-.69	-.86	-.94	-.11
Performance	-.31	-.56	.58	.64*
Redundancy index Predictor		12.79*		19.68*
variables (AP and LS)				
Preparing homework	-.11	-.46	.63*	.59*
Preparing for test	-.12	-.23	.33*	.63*
Preparing term papers	-.19	-.36	-.56	.09
Metacognitive self-regulation	-.99*	.76*	.08	-.34
Source management	-.65	-.15	-.79	-.72
Effort regulation	-.11	-.09	-.001	-.17
Cooperative in learning	-.01	-.12	.12	-.23
Help seeking	-.17	-.13	.18	-.29
Redundancy index		2.10*		2.23*
Canonical correlation coefficient		.41*		.34*
Canonical R^2^		.16		.12

* Coefficients with effect sizes larger than 0.3 (13).

## Discussion

 The purpose of this study was to examine the relationship between academic procrastination, learning strategies and statistics anxiety among Iranian college students. Our result shows that students with higher anxiety statistic would have the following combination of higher level of preparing homework (with canonical loadings= **-**0.46), preparing term papers (with canonical loadings= **-**0.36), and metacognitive self-regulation (with canonical loadings= 0.76). In other words, Procrastination and self-regulation all jointly and positively predict interpretation anxiety and test and class anxiety.

There are many reasons underlying the behavior of procrastination negatively affecting university life like all other areas of our lives. According to the studies regarding tendency to procrastination, the reasons were listed as poor time management skills, self-efficacy beliefs, discomfort regarding tasks, inability to concentrate, fear of failure, inability to orient objectives of success, anxiety, problem-solving skills, and working habits (16).

According to Rosário et al (15), the levels of academic procrastination increased as the students advanced throughout their educational process, and consequently did the underachievement. Albeit indirectly, these data indicate that adolescents usually put off difficult and unpleasant tasks that involve high doses of effort and may then generate anxiety (17,18), preferring to engage in activities that are more interesting to them (15). Their perception of task difficulty may be a significant factor that contributes to increasing procrastination behaviors.

Procrastination gets in the way of asking for help, possibly because of a fear on the students' part of exposing the fact that he has not been keeping up with assignments or readings. This is in turn negatively related to organization, an active, effortful learning strategy that results in the learner being closely involved in the task (9).

If a student has not been keeping up with his assignments or reading, he may likely use rehearsal and organization strategies as an ineffective means of trying to catch up, resulting in higher levels of anxiety when understanding concepts in class and on tests. With these findings, one might suggest that for men with statistics anxiety, assisting them with strategies addressing procrastination might be helpful. Such things as daily class assignments or in-class assignments may help them stay current in their study habits, making them less reliant on ineffective learning strategies and more open to asking for help when it is needed.

Among all of the learning strategies that have been investigated in relationship to academic procrastination, metacognitive self-regulation has received the most attention. Many studies found that students are less inclined to take risk, study ineffectively, memorize details and have poorer performance when they are highly anxious. For these reasons, anxiety is believed to be negatively related to self-regulate learning (2).

 In the second function also, it appears that for women, learning strategies like source management, rehearsal, organization, and elaboration can have positive effects on reduction statistics anxiety. Consistent with past research (6), all of the other learning strategies especially metacognitive self-regulation and source management are negatively related to statistics anxiety. I n other words, women appear to be better able at utilizing a wide range of learning strategies and this utilization influences their anxiety significantly. The second function for women closely resembles the findings for men: procrastination, rehearsal, organization, and elaboration is positively related to interpretation and test and class anxiety.

Although there is limited research in this area, it is plausible that the introduction of learning strategies may alleviate statistics anxiety since they provide a basis for modifying thought and behavioral habits. Motivational orientations and learning strategies, such as source management and metacognitive self-regulation, may serve as a form of cognitive restructuring. As a result, learning strategies may lead to a re-labeling of feelings and thoughts about statistics ultimately forming a new set of arousal cues that support the critical role of the neurological constructs that mediate anxiety.

 As in all studies, this study entails several limitations and these must be acknowledged.

 In terms of internal validity, procrastination was assessed via a self-report instrument that may have produced socially desirable responses. In future, the present study should be replicated using behavioral measures of variables in addition to, or instead of, self-report instruments.

With respect to external validity, the sample size was small, and so the findings cannot be generalized to the wider tourist population and to other product categories. Further studies should investigate the nature of this relationship using different products across different industries.

## Authors' contributions

ShV conceived and designed the evaluation, helped to the interpretation of the data, statistically analyzed the data, and contributed to draft the manuscript. FF participated in collecting the data and drafting the manuscript. FG also took part in data collection and evaluating them. AI helped to conceive and design the study and helped to the interpretation of the results. All authors read and approve the final manuscript.

## Notes

**Declaration of interest:** None.
